# Computational optical imaging with a photonic lantern

**DOI:** 10.1038/s41467-020-18818-6

**Published:** 2020-10-15

**Authors:** Debaditya Choudhury, Duncan K. McNicholl, Audrey Repetti, Itandehui Gris-Sánchez, Shuhui Li, David B. Phillips, Graeme Whyte, Tim A. Birks, Yves Wiaux, Robert R. Thomson

**Affiliations:** 1 Institute of Photonics and Quantum Sciences, Heriot-Watt University, Edinburgh, EH14 4AS UK; 2 EPSRC IRC Hub, MRC Centre for Inflammation Research, Queen’s Medical Research Institute (QMRI), University of Edinburgh, Edinburgh, UK; 3 Institute of Sensors, Signals and System, Heriot-Watt University, Edinburgh, EH14 4AS UK; 4 Department of Actuarial Mathematics and Statistics, Heriot-Watt University, Edinburgh, EH14 4AS UK; 5 Department of Physics, University of Bath, Claverton Down, Bath, BA2 7AY UK; 6 Wuhan National Laboratory for Optoelectronics, Huazhong University of Science and Technology, 430074 Wuhan, Hubei China; 7 School of Physics and Astronomy, University of Exeter, Exeter, EX4 4QL UK; 8 Institute of Biological Chemistry, Biophysics and Bioengineering, Heriot-Watt University, Edinburgh, EH14 4AS UK; 9 Present Address: ITEAM Research Institute, Universitat Politècnica de València, 46022 Valencia, Spain

**Keywords:** Fibre optics and optical communications, Imaging and sensing, Imaging techniques

## Abstract

The thin and flexible nature of optical fibres often makes them the ideal technology to view biological processes in-vivo, but current microendoscopic approaches are limited in spatial resolution. Here, we demonstrate a route to high resolution microendoscopy using a multicore fibre (MCF) with an adiabatic multimode-to-single-mode “photonic lantern” transition formed at the distal end by tapering. We show that distinct multimode patterns of light can be projected from the output of the lantern by individually exciting the single-mode MCF cores, and that these patterns are highly stable to fibre movement. This capability is then exploited to demonstrate a form of single-pixel imaging, where a single pixel detector is used to detect the fraction of light transmitted through the object for each multimode pattern. A custom computational imaging algorithm we call SARA-COIL is used to reconstruct the object using only the pre-measured multimode patterns themselves and the detector signals.

## Abstract (web summary)

Here, the authors demonstrate a route to high resolution microendoscopy using a multicore fibre with a photonic lantern. They show that distinct multimode patterns of light can be projected from the output of the lantern by individually exciting the single-mode MCF cores, whose patterns are highly stable to fibre movement.

## Introduction

Endoscopes that use bundles of optical fibres to transmit light in a spatially-selective manner have had a profound impact on minimally-invasive medical procedures. To reduce the device size and increase imaging resolution, this concept has been extended to individual fibres containing thousands of light-guiding cores. These single-fibre coherent fibre bundles (SF-CFBs) can provide resolutions of a few microns in the visible^1^. When combined with fluorescent contrast agents, they facilitate observation of disease processes at the cellular level^2^.

SF-CFBs are not without drawbacks. To maintain spatially-selective transmission of light, the fibre cores must be sufficiently spaced to keep core-to-core crosstalk at an acceptable level, intrinsically limiting imaging resolution and throughput. Recently, it has been shown that a highly scattering material can be placed onto the end of the SF-CFB to convert each single mode into a multimode pattern of light, and that these patterns can be used for compressive fluorescence imaging^3^. Unfortunately, the use of such a highly scattering material between the object and the distal end of the fibre will dramatically reduce the fluorescence collection efficiency—a key parameter that must be maximised for real-world in-vivo applications. The significant drawbacks of SF-CFBs has led to an explosion of interest in multimode fibre (MMF) imaging, where image information is carried by multiple overlapping spatial modes guided by one multimode core, rather than the many spatially separated cores of the SF-CFB. MMF imaging can deliver an order of magnitude higher spatial resolution, but it is far from trivial to implement because the amplitudes and phases of the MMF modes become scrambled along the fibre. This can be addressed by characterising the MMF’s transmission matrix and controlling a spatial light modulator to “undo” the scrambling^4,5^, but any movement of the fibre changes its transmission matrix, and access to the in vivo distal end is required for recalibration unless the new path is precisely known^6^.

One technology that could provide of a route to high resolution single-fibre microendoscopy is the multicore fibre (MCF) “photonic-lantern” (PL)^7^. PLs are guided-wave transitions that efficiently couple light from *N*_s_ single mode cores (the MCF) to a multimode waveguide like an MMF. PLs can be made by tapering (heating and stretching in a small flame) a single MCF^8^, such that the entire reduced-diameter MCF acts as the multimode end of the PL. *N*_p_ = *N*_s_ distinct multimode patterns of light are generated at the multimode output by coupling light into each core at the MCF input, one at a time. If the MCF exhibits negligible crosstalk between the cores along the length of the MCF, such that the light propagates along just one core, these patterns do not change when bending the fibre, unlike those of an ordinary MMF. This is because deformation of the MCF merely changes the overall phase of the output pattern. Unlike the spatially-separated modes of a SF-CFB (but like an ordinary MMF), the PL allows the full area of the fibre end-facet to be sampled, and the size of the patterns can be reduced to the minimum allowed by the numerical aperture (NA) of the multimode end.

Here, we demonstrate the feasibility of PL based microendoscopy by using a PL to implement a form of “single-pixel” imaging^9^ that we call computational optical imaging using a lantern (COIL). Light patterns generated by the PL are projected onto an object (e.g., tissue). Light returned from the object (e.g., fluorescence) is detected by a single-pixel detector, which for the microendoscopy application could be placed at the proximal end of the MCF. In this case, the single-pixel detector would measure the total returned signal across all MCF cores. Information about the distribution of light across these cores is not needed, and the manner in which the light is detected is not critical—as long as it is unchanged between calibration and measurement, and the proximal-end detection is not selecting part of an interference pattern due to waves from multiple cores (which would be sensitive to phases). The known patterns and measured return signals provide information about the object, from which an image can be formed^9^. We note that both Mahalati at al.^10^ and Amitonova et al.^11^ have demonstrated compressive single MMF imaging with proximal end signal detection, but we highlight that both suffer from the stability issues that MCF PLs address. We show that the quality and detail of the computed image can be greatly improved by exploiting an advanced image formation algorithm, that combines the measurement data with a generic prior postulating that the spatial structure of the image is underpinned by a small number of degrees of freedom. We demonstrate that COIL opens a promising route to efficient and practical high-resolution microendoscopy.

## Results

### Computational imaging algorithm

The starting point for our image reconstruction algorithm is to approach PL based imaging in the context of the theory of compressive sampling. In this context, one assumes that the image under scrutiny is sparse in some transform domain linearly related to the pixel domain (e.g., the domain of a wavelet transform^12^), that is to say that its spatial structure is underpinned by a small number of degrees of freedom. The sparsity prior information is leveraged to enable the image recovery from incomplete data. Compressive sampling approaches have been developed in a wide variety of imaging applications ranging from magnetic resonance imaging^13,14^, and astronomical imaging^15,16^, to ghost imaging^17,18^ and speckle imaging^19^. Optimisation algorithms represent the dominant class to solve inverse problems for image recovery from incomplete data. The image estimate is defined as a minimiser of an objective function, consisting of the sum of a data-fidelity term and a sparsity-promoting prior term. The resulting minimisation problem is solved through iterative algorithms progressively minimising the objective function.

From the perspective of the reconstruction algorithm, we choose to work in a regime where the number of pixels in the reconstruction is considerably higher than the number of patterns projected and their transmission values e.g., for reconstructions formed using 121 projected patterns, our reconstructions are 125 × 125 pixels in size (see Supplementary Note 1 in the Supplementary Information (SI) for a detailed description of the reasoning involved in setting the number of pixels in each reconstruction presented in this manuscript.). As we demonstrate through simulations, this regime is of particular interest for COIL as it can, in the future, allow the reconstruction of high resolution images without unrealistic demands on the number of MCF cores. Due to the high number of pixels in the reconstruction compared to the number of projected patterns, the inverse problem becomes heavily ill-posed and image formation requires strong prior information. With that aim we resort to an advanced “average sparsity” model firstly introduced in astronomical imaging^16^, where multiple wavelet transforms are introduced simultaneously to promote sparsity and reduce the effective number of degrees of freedom well-below the image size.

To solve the resulting minimisation problem, we rely on modern “proximal splitting” optimisation algorithms^20,21^ whose main features are a guaranteed fast convergence and low computational complexity. These algorithms have been used in computational imaging in a variety of fields (see ref. ^20^ and references therein). Building on the “average sparsity” approach we developed a proximal algorithm for COIL, dubbed sparsity averaging reweighted analysis (SARA)–COIL. Details of our optimisation approach are provided in the “Methods” section, together with a description of the associated MATLAB toolbox.

### Experimental techniques and results

**Fig. 1:**
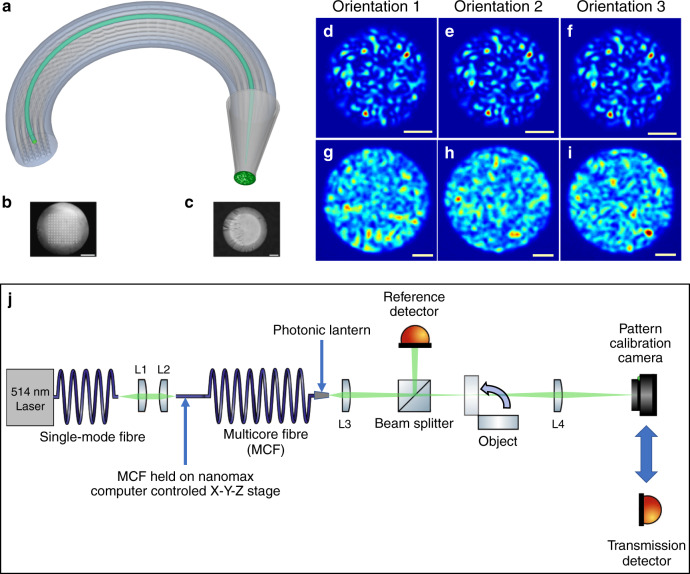
Computational imaging using a photonic lantern. **a** Schematic *N*_s_ = 25 square-array multicore fibre with a photonic lantern at one end. (in green) Light in one core excites a fixed light pattern at the lantern’s output. **b** Optical micrograph of the facet of the *N*_s_ = 121 multicore fibre used in this work. Scale bar: 50 μm. **c** Optical micrograph of the multimode output of the photonic lantern. Scale bar: 10 μm. **d**–**f** Near field intensity patterns at the output of the photonic lantern when one core of the multicore fibre is excited with monochromatic light (*λ* = 514 nm). The patterns are insensitive to fibre bending as shown by the micrographs obtained for three arbitrary conformations of the fibre. Scale bars: 10 μm. **g**–**i** Near field intensity patterns at the output of a 105 μm core multimode fibre when excited with monochromatic light (*λ* = 514 nm). As shown in the micrographs obtained for three arbitrary conformations of the fibre, the patterns are highly sensitive to bending of the fibre. Scale bars: 20 μm. **j** Experimental setup used to acquire the data during the photonic lantern imaging experiments. Full details of the data acquisition procedure are given in the “Methods” section.

Figure 1a is a schematic of an MCF (with *N*_s_ = 25 for clarity) with a PL at one end. For the work reported here, the PL was fabricated at one end of ~3 m of MCF with *N*_s_ = 121 single-mode cores in a 11 × 11 square array (Fig. 1b) with negligible core-to-core crosstalk at 514 nm. The multimode output end of the PL had a core diameter of ~35 μm and an NA of ~0.22 (Fig. 1c). See “Methods” section for fabrication details of the PL. Using computer-controlled alignment, each MCF core could be individually excited using coherent 514 nm laser light, generating *N*_p_ = *N*_s_ = 121 different multimode patterns of light at the output. Each output pattern was highly stable regardless of the conformation of the MCF, Fig. 1d–f. (See the “Methods” section for full details of how the stability of these patterns was quantified, together with Supplementary Fig. 1 for a schematic of the experimental setup used to characterise the pattern stability. Supplementary Figs. 2 and 3 present the patterns generated under different MCF deformation and excitation conditions. Supplementary Table 1 presents quantitative evaluations of the pattern stability under different MCF deformation and excitation conditions). This is due to the short length (~4 cm) of the PL transition itself and the minimal crosstalk between the MCF cores. In contrast, similar bending of an ordinary MMF changes the output pattern (Fig. 1g–i).

Our experimental setup for single pixel imaging (Fig. 1j) is similar to the computational ghost imaging system presented in the ref. ^18^, where a spatial light modulator projected random patterns of light onto a test object and detectors measured the fraction of power transmitted through the object. In our experiment the spatial light modulator was replaced with the PL, allowing *N*_p_ = *N*_s_ = 121 different patterns to be projected onto the object by exciting each core of the MCF individually. The experimental data acquisition procedure consisted of two steps. First, a camera was used to record the patterns generated in the object plane by exciting each of the cores individually. In the second, the object was moved into the beam path and a detector was used to record the magnitude of the light transmitted through the object. These pairs of data (patterns + transmission values) are the data used with the SARA–COIL algorithm (further details of the experimental setup and data acquisition procedure are presented in the “Methods” section). We highlight the fact that the MCF was intentionally moved and deformed significantly between pattern calibration and imaging experiments, to further highlight the stability of the PL approach.

**Fig. 2:**
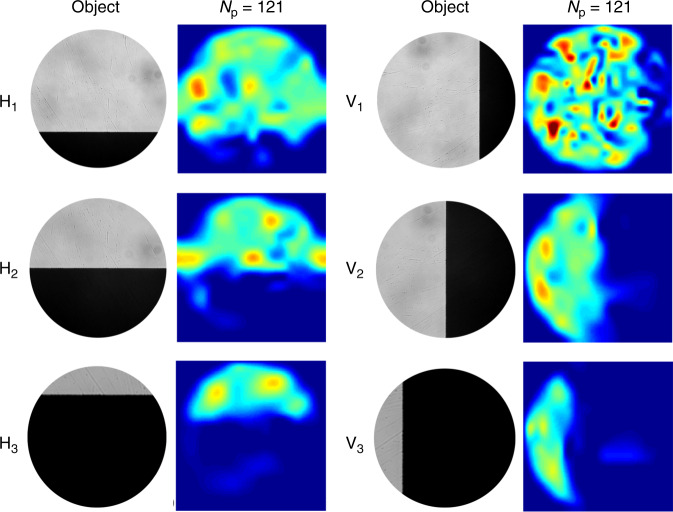
COIL imaging of a knife edge using experimental data. SARA–COIL results obtained using *N*_p_ = 121 patterns. Micrographs of the objects are shown in the first and third columns, while corresponding SARA–COIL reconstructions are presented in the columns to the right of each set of objects. *H*_i_ and *V*_i_ respectively denote objects formed by horizontally and vertically overlaying a knife edge over ~25% (*i* = 1), ~50% (*i* = 2), and ~75% (*i* = 3) of the intensity pattern. Each reconstructed image has 125 × 125 pixels, with a field of view in the object plane of 0.9 mm × 0.9 mm.

Initially, we used a simple “knife-edge” as the object. As shown in the object images of Fig. 2, the knife-edge was orientated either horizontally (H) or vertically (V) and positioned to block ~25, ~50, or ~75% of the pattern projected onto it. As shown in Fig. 2, COIL successfully reconstructs images of 125 × 125 pixels using only *N*_p_ = 121 patterns. All reconstructions we report using experimental data represent a 0.9 mm × 0.9 mm field of view at the object plane, where the lantern output is imaged with a magnification of ~26 for the purposes of this demonstration. Since the illumination light originates from the lantern itself, the resolution of a near-field imaging modality without the imaging optics would scale by the inverse of the same magnification.

**Fig. 3:**
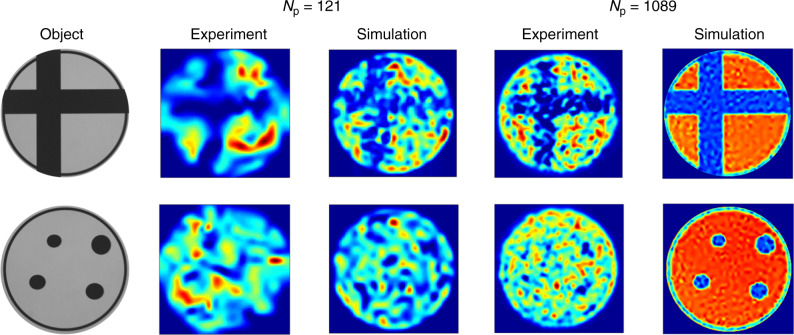
COIL imaging of objects using experimentally measured and simulated data. SARA–COIL reconstructions are presented using either *N*_p_ = 121 or *N*_p_ = 1089 patterns, and either experimentally measured or simulated pattern and overlap data. The objects are an off-centre cross and four asymmetrically-positioned elliptical dots, micrographs of which are presented. The reconstructions are either 125 × 125 pixels in size for the *N*_p_ = 121 case, or 377 × 377 pixels in size for the *N*_p_ = 1089 case. Reconstructions using simulated patterns and overlap data for the *N*_p_ = 121 case used patterns generated from random orthonormal superpositions of the 121 lowest order modes of a circular ideal-mirror waveguide. For reconstructions using *N*_p_ = 1089, the object was rotated about the optical axis by 320° in steps of 40°, effectively creating a total of 121 × 9 patterns. The field of view of all reconstructions using experimentally measured data is 0.9 mm × 0.9 mm in the object plane.

To confirm that COIL is applicable to more complex objects, we repeated the experiment using the objects shown in Fig. 3: an “off-centre cross” and “4 dots” positioned asymmetrically. SARA–COIL can clearly reconstruct the off-centre cross, further confirming the generality of the approach, but cannot reconstruct the small features in the “4-dots” object. To demonstrate how the imaging quality might improve by using an MCF PL with more cores, we repeated the data acquisition nine times with the object rotated by 40° between each, acquiring transmission data for each object using effectively *N*_p_ = *N*_s_ × 9 = 1089 different patterns. As expected, increasing the number of patterns significantly increases image quality for the off-centre cross (Fig. 3). It also reconstructs some features of the “4 dots” object but falls short of fully resolving them.

The reconstructions we have presented using experimentally acquired data are a proof-of-concept of the COIL approach, but the quality of the imaging we have obtained is limited and does not yet demonstrate a compressive advantage. As we discuss later, we believe this is primarily due to limitations with the current experimental setup. To investigate the imaging quality that could be achievable in the future using an optimised experimental system, we have performed detailed end-to-end simulations. To simulate the intensity patterns from an ideal *N*_s_ = 121 PL, we first calculated the field distributions of the 121 lowest-order spatial modes of a circular ideal-mirror waveguide. We then generated a set of 121 mutually-orthonormal but otherwise random coherent superpositions of the modes, and formed intensity patterns by taking the square modulus. We highlight that previously reported characterisation results have confirmed the adiabatic nature of our MCF PL transitions^22^, supporting the relevance of the PL pattern simulations to our experimental system. The imaging experiment was simulated by computing the overlap integral between each intensity pattern and the object. The intensity patterns and overlap data were then processed using SARA–COIL to reconstruct an image. The simulated reconstructions for both objects, using either *N*_p_ = 121 (not rotated) or *N*_p_ = 1089 (nine rotations), are shown in Fig. 3 alongside the reconstructions based on experimental data for comparison. As expected, images obtained using both measured and simulated data improved considerably as the number of patterns is increased. Furthermore, if we consider that the multimode port of the PL used in our experiments has a diameter of 35 μm, our *N*_p_ = 1089 simulations suggest that sub-micron resolution could be achievable using a PL generating only a thousand patterns. (The NA of the port would have to be ~0.3 to support this number of modes, rather than the 0.22 of the PL used here).

**Fig. 4:**
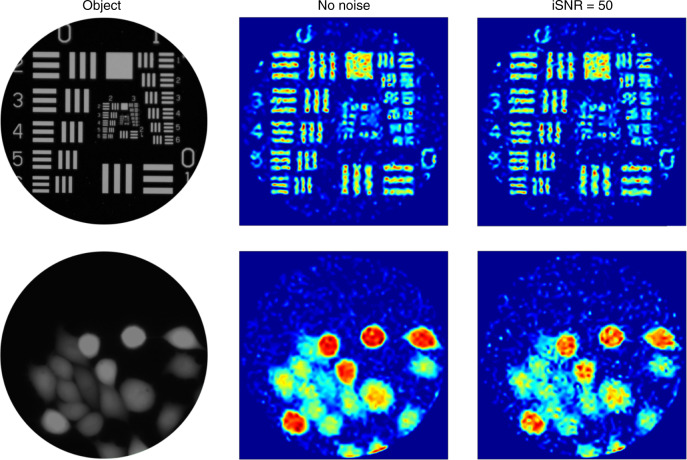
Simulations of COIL imaging with a high core count photonic lantern. Simulated reconstruction results (511 × 511 pixels) obtained using *N*_p_ = 2000 intensity patterns generated from random orthonormal superpositions of the 2000 lowest-order modes of a circular ideal-mirror waveguide. The objects were the 1951 USAF resolution target and a confocal microscope image of fixed calcein stained adenocarcinomic human alveolar basal epithelial (A549) cells. For each object, the reconstructed image with additive Gaussian noise (input signal-to-noise ratio iSNR = 50) is shown alongside that with no added noise. We highlight the fact that there is deliberately no spatial scale for the reconstructions, since the size of a waveguide supporting *N*_p_ = 2000 modes varies depending on its core-cladding refractive index contrast. The reader is referred to the discussion section for more information. We thank Eckhardt Optics for allowing us to use their image of the USAF 1951 resolution test chart presented in the top left.

To further highlight the potential of COIL for the high-resolution imaging of structures in vivo, we simulated (as above) the results that might be expected using a *N*_s_ = 2000 PL to project *N*_p_ = 2000 patterns. The two objects used for this simulation were an image of the 1951 USAF resolution target and a confocal microscope fluorescence image of fixed calcein-stained adenocarcinomic human alveolar basal epithelial (A549) cells. Our images, shown in Fig. 4, are high-quality reconstructions of both objects. Figure 4 also shows that our image reconstruction technique is robust to the presence of additive Gaussian noise in the overlap data. For example, both contrast and resolution are only minimally affected by the noise, and features such as the horizontal and vertical bars in the top right of the USAF target are still clearly resolvable.

**Fig. 5:**
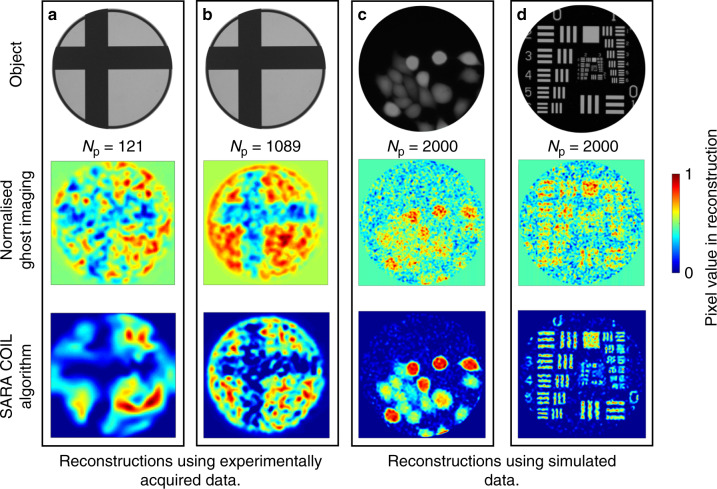
Comparing reconstruction algorithms for COIL applications. Reconstructions of various objects using experimental or simulated data and either an established ghost imaging algorithm (Eq. 5 in [Sun^18^]) (middle row) or SARA–COIL (bottom row). **a** 125 × 125 pixel reconstructions of an off-centre cross for *N*_p_ = 121 using experimental data. **b** 377 × 377 pixel reconstructions of an offset cross for *N*_p_ = 1089 using experimental data. **c**, **d** 511 × 511 pixel reconstructions of the A549 cells (**c**) and the USAF target (**d**) for *N*_p_ = 2000 using simulated patterns and overlap data. Note that regions with no available information are treated differently by the two algorithms. As seen in the corners of all images, the ghost imaging algorithm assigns a mid-scale value, whereas SARA–COIL assigns a value of 0. In images reconstructed from experimental data, 1 represents the regions of highest transmission, and in those based on simulated data 1 represents regions of highest intensity. The field of view of all reconstructions using experimental data is 0.9 mm × 0.9 mm in the object plane. We thank Eckhardt Optics for allowing us to use their image of the USAF 1951 resolution test chart presented at the top of column **d**.

For completeness, Fig. 5 compares SARA–COIL to a simpler, more intuitive, reconstruction algorithm used for classical ghost imaging—see Eq. 5 in the ref. ^18^. This algorithm uses only the fractional transmission of the projected pattern to weight its contribution to the image reconstruction. No attempt is made to optimise this towards a realistic object using a prior. The comparison confirms that SARA–COIL significantly improves both resolution and contrast, revealing features that are otherwise barely or not visible. These results provide a compelling justification for the advanced algorithmic approach we adopted.

## Discussion

The reconstructions presented in Fig. 3 using 1089 patterns clearly indicate that although our experimental results broadly agree with simulations from ideal data, there is considerable potential for more accurate reconstructions. We highlight that the quality of the reconstructions using experimental data is degraded by the fact that the re-centring of the object onto the pattern after each rotation was only performed by eye, using a thin ring around the object to guide alignment. In fact, both reconstructions in Fig. 3 show hints of resolving this ring. This practical limitation can be readily resolved by adopting MCFs with more cores and not rotating them.

Remarkably, Fig. 4 demonstrates that, even in the presence of noise, a future *N*_s_ = *N*_p_ = 2000 COIL system could be capable of resolving objects separated by just ~1.6% of the multimode core diameter (see the three-bar pattern at the top right of the USAF target). If these objects were point-like objects, this would indicate the potential to resolve ~3000 point source objects across the fibre facet. This is a clear demonstration of the future potential advantage of using COIL in combination with the SARA–COIL algorithm for compressive optical imaging. To put this into a future use-case context, if a COIL system is constructed to operate using 488 nm excitation light and an *N*_s_ = 2000 MCF, the multimode output of the PL could have a 63 μm diameter core with an NA of 0.22, assuming established fabrication techniques^8^ with an F-doped silica cladding—see “Methods” section. Such a system could resolve objects separated by just ~1.25 μm. This is close to the 1.35 μm expected from Rayleigh’s criterion (0.61 *λ*/NA), a strong indication that COIL can deliver at least diffraction-limited imaging across the field of view of the core.

The *N*_s_ = *N*_p_ pattern projection is only the simplest imaging modality one might consider using PLs for. In fact, PLs could enable significantly more advanced and powerful modalities, some driven by compressive sampling principles, but these require the controlled simultaneous excitation of multiple MCF cores to generate coherent combinations of the multimode states at the output. To do this in a controlled manner, the key information to be obtained are the relative phases and amplitudes of the individual basis patterns at the multimode output. We envisage future COIL imaging systems exploiting polarisation maintaining MCFs, where the PL’s output is coated to partially reflect some pump or metrology light back along the MCF. Since each multimode pattern generates a specific nonbinary phase and amplitude distribution across the MCF cores after reflection, and since there is negligible crosstalk between the MCF’s cores, the distribution of reflected light across the cores at the proximal end will encode the relative phases and amplitudes of the multimode patterns at the output. In principle, this could facilitate the coherent synthesis of arbitrary excitation fields at the output of the lantern for both near-field and far-field spot-scanning modalities. To demonstrate the feasibility of these more advanced MCF PL-based imaging approaches, Supplementary Fig. 4 in the SI presents results of transmission matrix measurements, and coherent light control, to generate spot scanning at the multimode output of an integrated photonic lantern. Also see Supplementary Movie 1 in the SI to view the full transmission matrix characterisation, and Supplementary Movie 2 in the SI for a visualisation of the coherent generation of a scanning spot at the multimode end of the integrated lantern. Further details of these preliminary experiments on coherent mode combination in a photonic lantern are given in Supplementary Note 2 in the SI. Such coherent mode combination approaches could also enable the projection of many more than *N*_s_ different known multimode patterns. As detailed by Mahalati et al.^10^, the number of possible “intensity modes”, and therefore the number of resolvable features across the output core, could reach a maximum of *4N*_s_. For the case of an *N*_s_ = 2000 PL with a 63 μm diameter 0.22 NA multimode core operating at 488 nm, such an approach could deliver a resolution of ~626 nm—significantly smaller than the Rayleigh limit and opening a potential route to super-resolution microendoscopy. The NA of the PL’s multimode output can also be pushed well beyond 0.22 by exploiting more advanced fibre approaches. For example, we foresee the creation of PL’s using a polarisation maintaining MCF with a double-cladding geometry, such as those commonly used in fibre lasers for efficient cladding pumping. In this case, the MCF cores and their glass cladding would be surrounded by an air cladding that could facilitate a PL multimode port at the distal end with an in vivo NA of up to ~0.65 at 488 nm^23^. This might deliver a spatial resolution of ~212 nm, although stability issues during in vivo exposure will obviously play a role in determining this.

We resorted to a powerful framework of optimisation to develop the SARA-COIL algorithm, but further developments may significantly improve image estimation. Firstly, regularisation priors specifically developed for images of interest in microendoscopy can improve quality over our state-of-the-art “average sparsity” prior. Secondly, parallelised “proximal algorithms”^24,25^ can improve scalability to high-resolution imaging, ultimately to provide real-time microendoscopic imaging. Finally, approximation in the measurement model can severely affect imaging quality in computational imaging (e.g., the alignment between object and patterns). Joint calibration and imaging algorithms can be defined in the theory of optimisation, that can simultaneously solve for unknown parameters in the measurement model and form the image^26,27^.

To conclude, we have experimentally demonstrated a form of single-pixel imaging using a multicore fibre and photonic lantern to generate distinct multimode light patterns. We have provided compelling evidence that this, underpinned by the powerful SARA-COIL optimisation algorithm, can deliver at least diffraction-limited imaging across the full area of a multimode fibre core, without sensitivity to bending or any need to control or compensate for modal phases. This meets the world-wide need to develop new fibre-optic imaging techniques to deliver high-resolution images of cellular and molecular mechanisms in vivo. We have also discussed how it opens a route to more complex imaging modalities, such as super-resolution microendoscopy with submicron resolution. We also anticipate that COIL could also be useful in applications that benefit from a reduced number of measurements, such as fibre-optic epifluorescence or confocal microendoscopy, which are vulnerable to detrimental effects such as photobleaching and phototoxicity^28^.

## Methods

### The multicore fibre

The *N*_s_ = 121 square-array multicore fibre was originally fabricated for a study of wavelength-to-time mapping^22^. The cores were positioned on a square grid with a core-to-core spacing of ~10.53 µm. The mode field diameters of the MCF cores were measured at 514 nm using calibrated near-field imaging. The 1/*e*^2^ mode field diameter was ~2.1 ± 0.2 μm.

### Photonic lantern fabrication

To fabricate the PL^8^, the MCF was threaded into a fluorine-doped silica capillary, the refractive index of which is lower than the pure silica cladding of the MCF. The capillary was collapsed, by surface tension, on top of the MCF using an oxybutane flame. Using a similar flame, the cladded structure was then softened and stretched by a tapering rig, forming a biconical fibre-like structure. The multimode port of the PL was finally revealed by cleaving the centre of the tapered waist. The resultant multicore-to-multimode taper was ~4 cm long, with an approximately linear profile. The multimode port’s core diameter was ~35 µm and its numerical aperture was 0.22.

### Quantifying the stability of the multimode patterns

Supplementary Fig. 1 shows the experimental setup we used to characterise the stability of the patterns generated at the multimode end of an MCF lantern. Light from a 514 nm diode laser was transported to the MCF using a single mode fibre (SM450 from Thorlabs, single mode at 514 nm, NA between 0.1 and 0.14) and coupled into the MCF using direct fibre-to-fibre coupling. By minimising the gap between the single mode fibre and the MCF it was possible to excite any core of the MCF individually. By increasing the gap slightly, it was possible to excite multiple cores simultaneously. Light emerging from the multimode end of the photonic lantern was focused using a lens onto a CMOS camera, and near-field images of the multimode output could be digitally captured using a computer. To investigate the stability of the multimode patterns we captured images of the patterns under the following excitation conditions.

(i) Characterising the effect of excitation polarisation: to investigate the effect of laser polarisation on the multimode patterns, five images of the multimode output were captured. During the data acquisition, the MCF was not disturbed but the single mode fibre delivering light from the laser to the MCF was either left undisturbed or wrapped around a 25 mm diameter circular rod one, two, three, or four times to alter the excitation polarisation in a random manner.
(ii) Characterising the effect of MCF deformation: to investigate the effect of deforming the MCF, five images of the multimode pattern were captured. During the data acquisition, the single mode fibre delivering light from the laser to the MCF was not disturbed, but the MCF was either left undisturbed or wrapped around a 25 mm diameter circular rod either one, two, three, or four times. This entire procedure was repeated five times, each time coupling light into a different core individually. (The cores were chosen and numbered arbitrarily.) The procedure was also repeated when coupling light into many cores simultaneously. Multicore excitation was achieved by coupling the excitation fibre to the middle of the MCF core array, and then increasing the gap between the excitation fibre and the MCF to ~500 μm. Given the NA of the excitation fibre (~0.1–0.14), we estimate that the spot size on the input the MCF will be in the region of ~50 μm. Given that the MCF cores are spaced by ~10.53 µm, we estimate that ~40 of the MCF cores were excited.
(iii) Characterising the measurement precision: light was coupled into one core and five images of the multimode output were captured in quick succession without moving either the MCF or the single mode fibre delivering laser light from the laser to the MCF. This assesses the limits of the measurement system, since the patterns being captured are identical.

The similarity in the multimode patterns for each situation described above was quantified by calculating the overlap integral for each pair-wise combination of the images in each data set. For our purposes, the overlap integral was defined as:

1 $${\mathrm{Overlap}}\;{\mathrm{integral}} = \frac{{\left( {{\int} {I_A \cdot I_B} } \right)^2}}{{{\int} {\left( {I_{\mathrm{A}}^2} \right){\int} {\left( {I_{\mathrm{B}}^2} \right)} } }},$$

where *I*_A_ and *I*_B_ are the intensity distributions of the two patterns. The denominator normalises the calculation such that the overlap is 1 when the two patterns being compared are identical.

Supplementary Figs. 2, 3 present the results of the pattern stability measurements. Each column presents the five images of the multimode pattern acquired under specific excitations and fibre (SMF or MCF) deformations.

Supplementary Table 1 presents a summary of the pattern stability measurements. The patterns generated when exciting more than one MCF core simultaneously are highly unstable, with an overlap of 0.779 ± 0.02. This is to be expected from the clear visual differences in the relevant images shown in Supplementary Fig. 2a, and is due to core-dependent bending-induced phase shifts across the MCF^29^. The situation is dramatically different when exciting only one core of the MCF at a time. Under this condition the patterns exhibit an overlap of 0.978 ± 0.009. The fact that this value is very close to 1, and within error of the 0.985 ± 0.003 measurement precision limit of our experimental system, confirms the extremely high degree of stability of the multimode patterns generated when exciting only one MCF core at a time. It is interesting to note that an overlap of 0.958 ± 0.012 was measured when varying the excitation polarisation by deforming the single mode excitation fibre. This value may indicate that changing the polarisation of the excitation light has a very slight effect on the generated patterns but raises the question as to why this is not seen when deforming the MCF. One possibility is that the MCF is more polarisation preserving than the single mode excitation fibre, so that deforming the MCF has a reduced impact on the output polarisation.

### Imaging data acquisition

Imaging data using the photonic lantern was acquired using the experimental system shown in Fig. 1j. This consisted of a 514 nm continuous wave laser source, the light from which was transported to the experimental setup using a single mode fibre. The light from the single mode fibre was collimated using a fibre collimation package (L1) and focused onto the proximal facet of the MCF using lens L2. The proximal end of the MCF (the end without the photonic lantern transition) was mounted on a computer controlled NanoMax six-axis translation stage which provided nanometre resolution and ~μm bi-directional repeatability in the positioning of the MCF relative to the excitation laser focus. The end of the MCF with the photonic lantern was mounted on a manual three-axis translation stage and its multimode facet was imaged to the object plane using lens L3. The object plane was then relay imaged onto a CMOS camera using lens L4. A fraction of the light from the photonic lantern was sent to a reference detector before the object, allowing instabilities in the laser and any variations in coupling efficiency to be accounted for during data acquisition.

The experimental procedure consisted of two steps. In Step 1, a CMOS camera was used to record the patterns generated in the object plane (without the object in place) by exciting each of the cores individually. During this step, the NanoMax positions were noted to allow each MCF core to be excited and addressed individually during the next step. In Step 2, the object was moved into the beam path at the object plane, the CMOS camera was replaced with the “Transmission detector” to record the magnitude of the light transmitted through the object. The NanoMax positions recorded in Step 1 were used to address and excite each core individually with ~μm precision. For each core, the throughput was maximised using the ~nm resolution movements of the computer-controlled stages. The magnitudes of the signals from both the reference detector and the transmission detector were then recorded for each of the patterns projected onto the object. In this manner, the overlap of each projected pattern on the object was measured. It took ~1 h to collect the data for 121 cores. This data, in combination with the recorded patterns, are the data used with the SARA–COIL algorithm.

### SARA–COIL algorithm

The observed data, denoted by $y \in {\Bbb R}^{N_{\mathrm{p}}}$ (there is one data point per pattern), consist of a linear transform of the image of interest $x \in {\Bbb R}^{\mathrm{n}}$ with a linear operator whose lines consist of the projection patterns. The measurement model thus reads:

2 $$y = {\Phi} x + e,$$

where ${\Phi} \in {\Bbb R}^{N_p \times n}$ represents the measurement operator and $e \in {\Bbb R}^{N_{\mathrm{p}}}$ the acquisition noise.

The SARA–COIL algorithm results from an adaptation of the “SARA” approach developed by Carrillo et al.^16^. On the one hand, the minimisation problem solved reads as

3 $${\mathrm{minimise}}\,\left\| {{\it{{\Omega} {\Psi} }}{\mathrm{x}}} \right\|_1{\mathrm{subject}}\;{\mathrm{to}}\;{\it{x}} \in \left[ {0, + \infty } \right[^n{\mathrm{and}}\left\| {{\it{y}} - {\it{{\Phi} x}}} \right\|_2 \le \epsilon .$$

The first element in this expression is the sparsity-promoting prior term to be minimised. ||.||_1_ denotes the nondifferentiable ℓ_1_ norm, traditionally invoked in the context of compressive sampling. ${\mathrm{{\Psi} }} \in {\Bbb R}^{L \times n}$ is the linear operator defining the sparsity transform, built as the concatenation of nine wavelet transforms (*L* = 9*n*) as in Carrillo et al.^16^. ${\mathrm{{\Omega} }} \in {\Bbb R}^{L \times L}$ is a diagonal weighting matrix computed using a reweighting procedure introduced by Candès et al.^30^. The second element of the expression “ $\in [0, + \infty ]^n$ ” is a prior term imposing the physical constraint of positivity of the intensity image to be formed. The third element “$y - {\Phi} x_2 \le {\it{\epsilon }}$” is the data-fidelity term imposing that the discrepancy between data and model is bounded by the noise energy *ϵ*.

To solve this minimisation problem, we developed an iterative algorithm based on the primal-dual forward-backward “proximal algorithm”^31,32^.

## Supplementary information

Supplementary Information

Description of Additional Supplementary Files

Supplementary Movie 1

Supplementary Movie 2

## Data Availability

Raw data will be made available through the Heriot-Watt University PURE research data management system. 10.17861/a1bebd55-b44f-4b34-82c0-c0fe925762c6.

## Code availability

A MATLAB toolbox gathering the algorithm implementation as well as the data necessary to reproduce our simulations results using the USAF resolution target is available on GitHub at https://basp-group.github.io/SARA-COIL/.
