# Supplementary material for: Computational optical imaging with a photonic lantern
Source: Nat Commun. 2020 Oct 15;11:5217. doi: 10.1038/s41467-020-18818-6 (PMC7562926; doi:10.1038/s41467-020-18818-6)
Supplement: Supplementary file 1 — Supplementary Information [file 41467_2020_18818_MOESM1_ESM.pdf]

Supplementary Information

Choudhury et al, "Computational optical imaging with a photonic lantern"

Supplementary Note 1: Setting the number of pixels in each reconstruction

As discussed by Mahalati et al [1], when using pattern projection computational imaging and nonlocal reconstruction, the number of resolvable objects across the facet of a multimode fibre can approach *4N*, where *N* is the number of modes supported by the fibre given by:

$$N = \left(\frac{\pi\, d\, NA}{2\, \lambda}\right)^2$$

Supplementary Equation 1

In our experiments, the NA of the multimode end of the MCF-lantern is 0.22, the core diameter is 35 μm, and the wavelength of operation is 514 nm. The maximum possible number of resolvable objects across the fibre facet would therefore be ~2,000. In reality, the number of objects our imaging system will be able to resolve will be considerably lower than this because the MCF has only 121 cores and the adiabatic nature of the MCF-lantern only allows us to excite the 121 lowest order modes of the multimode port.

For the *Np* = 121 experiments (Fig. 3), we chose to set the number of pixels in the reconstruction to be 15,625, approximately an order of magnitude higher than 2,000. Under such a situation, the pixelation of the reconstruction cannot, in any way, limit the imaging resolution of the reconstruction. For the *Np* = 1,089 experiments (Fig. 3), which increased the number of projected patterns by almost an order of magnitude through rotation of the object, we chose to also increase the number of pixels in the

reconstruction by approximately an order of magnitude to 142,129. The key point is that in both experimental cases the number of pixels in the reconstruction was chosen to be sufficiently high that pixelation cannot limit the achievable imaging resolution. For the reconstructions generated using simulated patterns and simulated overlap data, also presented in Fig. 3, we logically set the number of pixels in the reconstructions to be the same as the number of pixels in the reconstructions using experimentally measured data.

The reconstructions presented in Fig. 4 used Np = 2,000 simulated intensity patterns and 2,000 simulated overlap data. The work of Mhalati et al [1] would indicate we would be able to resolve 8,000 objects across the fibre, and so we set the number of pixels in the reconstruction to be 261,121, which is well over an order of magnitude higher. Again, the key point is that pixelation of the reconstruction does not, in any way, limit the imaging resolution obtained in the simulation.

Supplementary Note 2: Coherent mode combination in a photonic lantern for near-field spot-scanning

In the ideal case, a photonic lantern transition performs a unitary transformation on the optical modes, such that the light propagates between two orthogonal basis sets - the first being the modes of the minimally coupled single mode cores, the second being the spatially overlapped modes of the multimode port. The unitary nature of this transformation means that the individual complex patterns of light generated by exciting each single mode will also form a complete basis set. Thus, by controlling the amplitudes and phases of the light coupled into the single mode cores we anticipated

it should be possible to coherently synthesize arbitrary excitation fields at the output of the lantern for both near- and far-field spot-scanning modalities.

As a proof-of-principle of this basic capability, we performed preliminary experiments using an integrated photonic lantern fabricated using ultrafast laser inscription, similar to the device reported in [2]. This integrated photonic lantern was fabricated inside a Corning Eagle 2000 substrate using a 500 kHz train of ultrafast pulses (~350 fs duration, 84 nJ, ~1 μm wavelength) which were focused into the substrate using a 0.4 NA lens. The lantern was constructed from 25 individual waveguides, each of which was fabricated using the multiscan technique [3] by translating the substrate through the laser focus 18 times with an inter-scan separation of 200 nm and a translation speed of 4 $mm.s^{-1}$. At one end of the lantern, these building block waveguides were arranged into a 5 × 5 array of minimally coupled waveguides, with an inter-waveguide separation of 30 μm in both axis. To create the lantern transition, these waveguides were brought together along the 15 mm length of the substrate to form a single multimode waveguide with dimensions ~18 μm × 18 μm. The individual waveguides were designed and observed to be single mode at around 785 nm, and so were few-moded at the 633 nm wavelength used in the coherent mode combination experiments described in the following.

We used an experimental setup similar to that used in [4] to characterise the transmission matrix of the integrated photonic lantern. This involved placing the integrated photonic lantern in one arm of a Mach-Zhender interferometer which contained a digital micro-mirror device (DMD – Vialux V-7001 XGA) placed before the photonic lantern. The DMD can be controlled to exhibit binary reflection patterns that diffract a complex field into the first diffraction order. By blocking light around this order,

the DMD can be used as a complex beam shaping device capable of creating arbitrary amplitude and phase patterns. In this way, we used the DMD to raster-scan a focused beam across the individual input waveguides of the integrated photonic lantern. The mutlimode port of the lantern was re-imaged onto a camera, together with a reference beam from the second arm of the Mach-Zhender interferometer. To characterise the transmission matrix of the integrated photonic lantern, light was coupled into each input waveguide individually, Supplementary Fig. 4(a, c, e), and phase stepping holography was used to reconstruct the complex field of the multimode output facet, Fig. 4(b, d, f). Following characterisation of the transmission matrix, it could be inverted to retrieve the input field required to create a foci at target positions at the multimode output facet. The DMD generates the required input fields Fig. 4(g, i, k)., which are modulated to raster scan the coherently formed focus at the multimode output of the integrated photonic lantern. Supplementary Fig. 4 (h,j,l) shows examples of these foci at several different positions

The results in Supplementary Fig. 4 demonstrate how the two-dimensional intensity distribution of light at the multimode output of a photonic lantern can be controlled via the amplitudes and phases of the light used to excite the input waveguides. This work focused on demonstrating the potential of using coherent mode combination in photonic lanterns for future spot scanning system imaging applications, but future work will also focus on generating quasi-random patterns for single pixel imaging applications using non-local reconstruction algorithms such as SARA-COIL. Although this work used an integrated photonic lantern for convenience, there is no reason to believe our approaches would not be applicable to MCF-lanterns for future applications in microendoscopy. We note that movement of the MCF does affect the

relative phase of light propagating through each of the MCF cores, as demonstrated in Supplementary Fig. 2(a). In our current work using MCF-lanterns we are insensitive to these phase changes as we only make use of the intensity of the output patterns created by sequential excitation of individual cores. However, prospective future developments of photonic lantern imaging that rely on coherent control to scan focussed spots at the output will be sensitive to such bend induced phase changes. In that case it will thus be necessary to monitor and correct for these relative phase changes. We expect this to be a more tractable problem than in the case of multimode fibres, as bends induce only relative phase changes but negligible mode coupling. Therefore, we envisage that input light reflected back to the proximal end from a partially reflective coating applied at the multimode end of the photonic lantern will carry enough information to correct for bend induced phase changes in real-time [5]. We disclose that results similar to those presented in Supplementary Fig. 4 were also recently disclosed at the 2020 Conference on Lasers and Electro-Optics [6]"

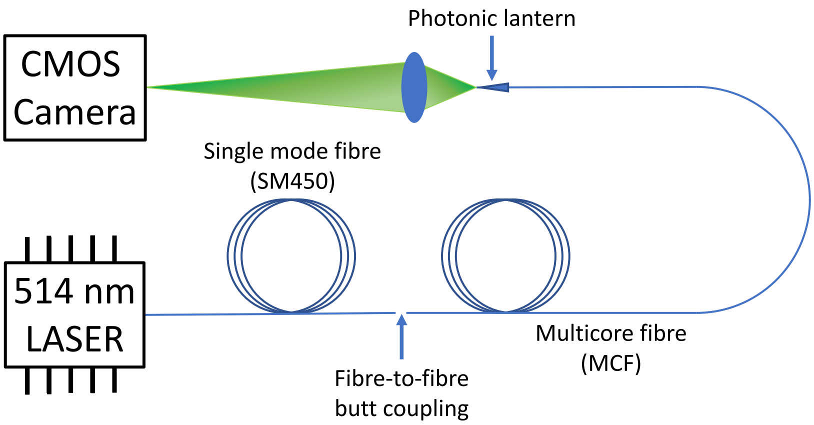

Supplementary Fig. 1: Pattern stability characterisation setup. Experimental setup used to characterise the stability of the multimode patterns generated at the multimode port of the photonic lantern.

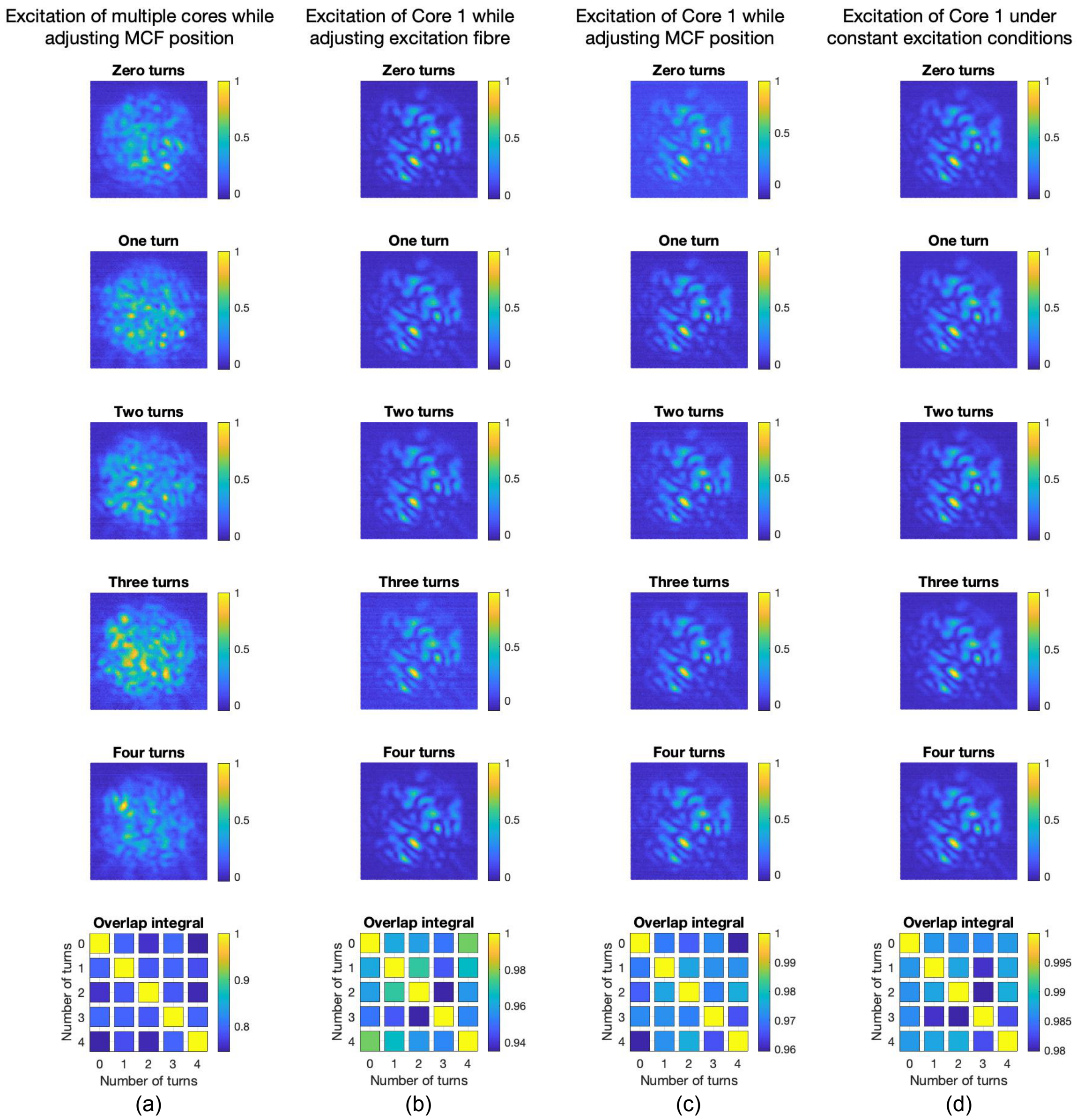

Supplementary Fig. 2: Pattern stability measurements obtained using either multi- or single-core MCF excitation. Each column presents 5 images of the multimode pattern at the output of the photonic lantern under specific excitations and fibre (SMF or MCF) deformations. Each "turn" corresponds to wrapping the relevant fibre around a 25 mm diameter pipe once. Column (a) was obtained when multiple cores of the MCF are excited simultaneously. Columns (b-d) were acquired when exciting only core 1. At the bottom of each column is the square matrix of overlap integrals (NB different scales), calculated using the overlap integral described in the "Quantifying the stability of the multimode patterns" section of the Methods, and the pair-wise combinations of the patterns in each data set.

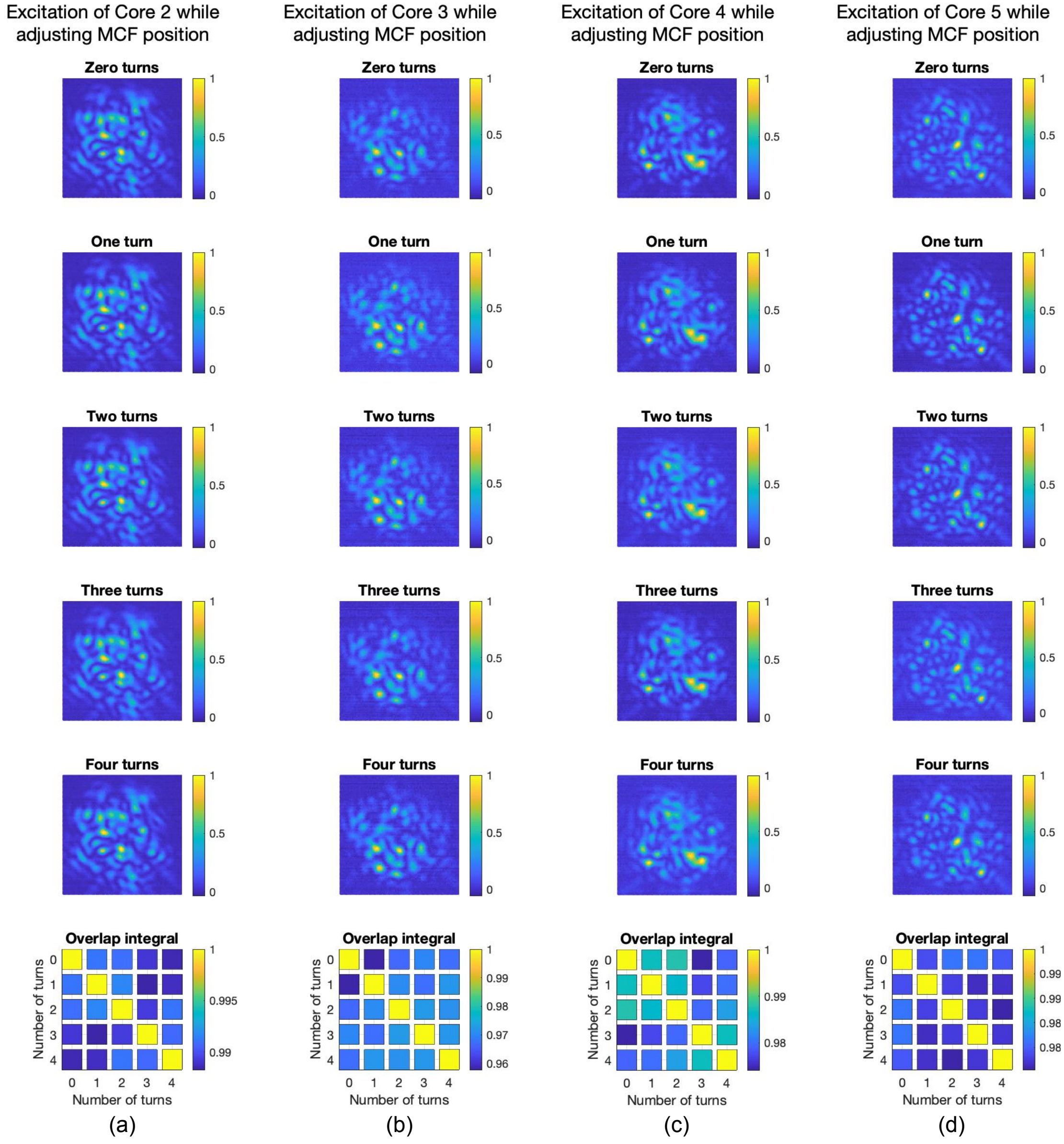

Supplementary Fig. 3: Pattern stability measurements obtained using single-core MCF excitation. Each column presents 5 images of the multimode pattern at the output of the photonic lantern under single-core excitation and different MCF deformations. Each "turn" corresponds to wrapping the MCF around a 25 mm diameter pipe once. At the bottom of each column is the square matrix of overlap integrals (NB different scales), calculated using the overlap integral described in the "Quantifying the stability of the multimode patterns" section of the Methods, and the pair-wise combinations of the patterns in each data set. Columns (a), (b), (c), and (d) present data acquired when exciting different MCF cores individually.

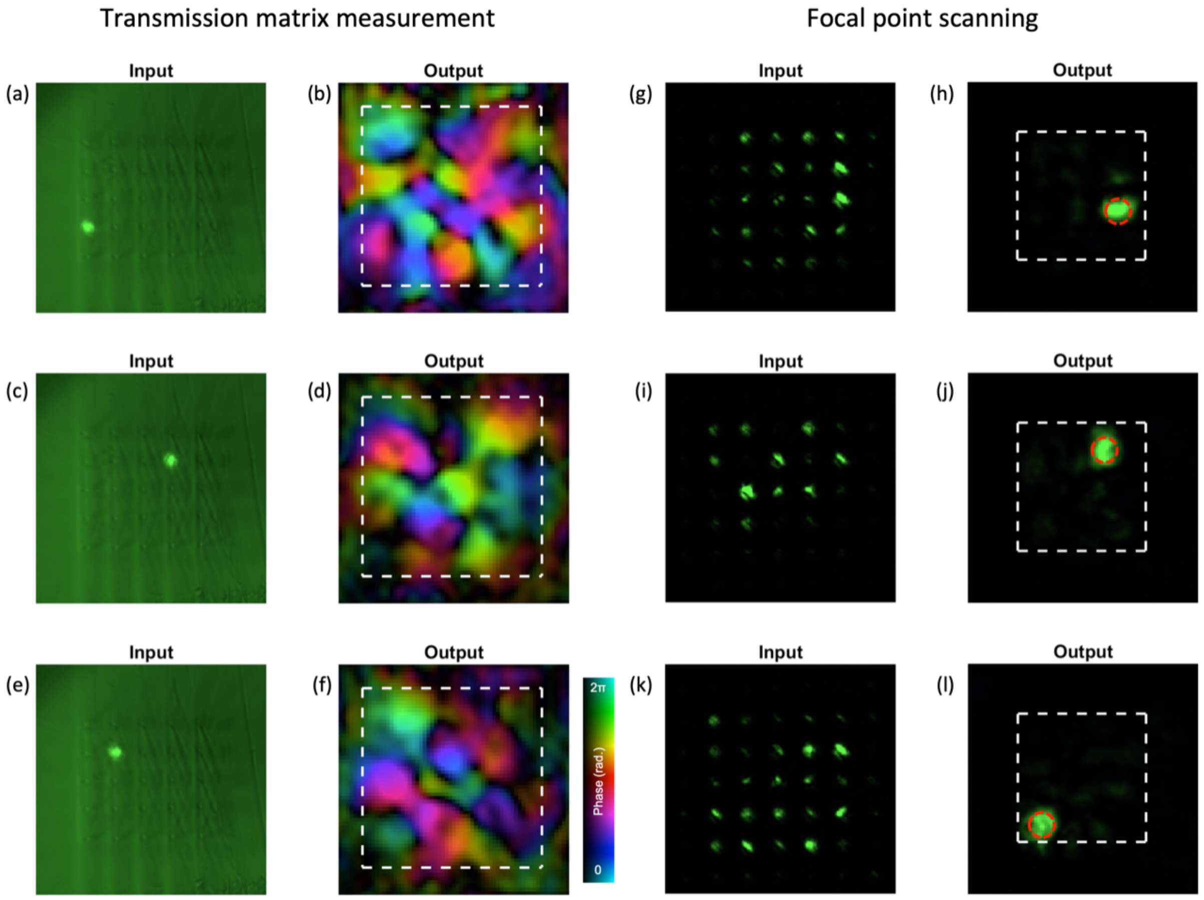

Supplementary Fig. 4: Coherent mode combination using an integrated photonic lantern. (Left two columns) Results of transmission matrix measurements. (a, c, e) show individual waveguides being excited, while (b, d, f) show the corresponding complex multimode fields at the output of the integrated lantern. (Right two columns) Coherent light control to generate spot scanning at the multimode output of the photonic lantern. (g, i, k) show multiple input waveguides being excited simultaneously, while (h, j, l) show the corresponding output from the multimode end of the lantern. Also see Supplementary Movie 1 to view the full transmission matrix characterisation, and Supplementary Movie 2 for a visualisation of the coherent generation of a scanning spot at the multimode end of the integrated lantern.

How was the MCF excited?	What was varied during the data acquisition?	Aim of data set	Number of unique pair-wise pattern combinations	Minimum overlap integral	Maximum overlap integral	Average overlap integral	Standard deviation of data
Multiple MCF cores excited simultaneously.	MCF path systematically deformed	To investigate how bend-insensitive the multimode patterns are when multiple MCF cores are excited simultaneously	10	0.745	0.800	0.779	0.02
Single MCF core excited.	No alterations	To determine the precision limit of the pattern stability measurement system	10	0.980	0.988	0.985	0.003
Single MCF core excited	Single-mode excitation fibre path systematically deformed.	To investigate how bend-insensitive the multimode patterns are when the excitation polarisation is varied.	10	0.935	0.977	0.958	0.012
Single MCF core excited	MCF path systematically deformed	To investigate how bend-insensitive the multimode patterns are when a single MCF core is excited.	50	0.958	0.992	0.978	0.009

Supplementary Table 1: Summary of pattern stability measurements

Supplementary References

[1] Mahalati, R. N., Yu, Gu R., and Kahn, J. M., "Resolution limits for imaging through multi-mode fiber," *Opt. Express* **21**, 1656-1668 (2013)

[2] Thomson, R. R., Birks, T. A., Leon-Saval, S. G., Kar, A. K. and Bland-Hawthorn, J. "Ultrafast laser inscription of an integrated photonic lantern," *Opt. Express* **19**, 5698-5705 (2011)

[3] Nasu, Y., Kohtoku, M. and Hibino, Y. "Low-loss waveguides written with a femtosecond laser for flexible interconnection in a planar light-wave circuit," *Opt. Lett.* **30**, 723-725 (2005)

[4] Turtaev, S., Leite, I. T., Mitchell, K. J., Padgett, M. J., Phillips, D. B., and Čižmár, T., "Comparison of nematic liquid-crystal and DMD based spatial light modulation in complex photonics," *Opt. Express* **25**, 29874-29884 (2017)

[5] Warren, S. C., Kim, Y., Stone, J. M. Mitchell, C., Knight, J. C., Neil, M. M. A., Paterson, C., French, P. M. W., and Dunsby, C., "Adaptive multiphoton endomicroscopy through a dynamically deformed multicore optical fiber using proximal detection," *Opt. Express* **24**, 21474-21484 (2016)

[6] Li, S., McNicholl, D. K., Whyte, G., Birks, T. A., Thomson, R. R. and Phillips, D. B., “Coherent control of light through laser written photonic lanterns” Technical digest of the Conference on Lasers and Electro-Optics (CLEO), Paper SM1L.4 (2020)
