# Supplementary material for: Computational optical imaging with a photonic lantern
Source: Nat Commun. 2020 Oct 15;11:5217. doi: 10.1038/s41467-020-18818-6 (PMC7562926; doi:10.1038/s41467-020-18818-6)
Supplement: Supplementary file 2 — Description of Additional Supplementary Files [file 41467_2020_18818_MOESM2_ESM.pdf]

Description of Additional Supplementary Files

File Name: Supplementary Movie 1
Description: Integrated lantern transmission matrix measurements. (Left) Movie showing the input to the integrated lantern, with the individual inputs excited in turn. (Right) Movie of the complex multimode output of the integrated lantern as each input to the lantern is excited. In this movie, intensity represents the strength of the electric field, while colour represents phase – see Supplementary Fig. 4.

File Name: Supplementary Movie 2
Description: Coherent mode combination in an integrated photonic lantern. (Left) Movie showing the input to the integrated lantern, with multiple individual inputs excited simultaneously. (Right) Movie of the multimode output of the integrated lantern as different input modes are coherently excited
